# Child mortality in England compared with Sweden: a birth cohort study

**DOI:** 10.1016/S0140-6736(18)30670-6

**Published:** 2018-05-19

**Authors:** Ania Zylbersztejn, Ruth Gilbert, Anders Hjern, Linda Wijlaars, Pia Hardelid

**Affiliations:** a The Farr Institute of Health Informatics Research, London, UK; b Population, Policy and Practice Programme, UCL Great Ormond Street Institute of Child Health, London, UK; c Children's Policy Research Unit, UCL Great Ormond Street Institute of Child Health, London, UK; d Centre for Health Equity Studies (CHESS), Stockholm University, Stockholm, Sweden; e Clinical Epidemiology Unit, Department of Medicine, Karolinska Institutet, Stockholm, Sweden

## Abstract

**Background:**

Child mortality is almost twice as high in England compared with Sweden. We aimed to establish the extent to which adverse birth characteristics and socioeconomic factors explain this difference.

**Methods:**

We developed nationally representative cohorts of singleton livebirths between Jan 1, 2003, and Dec 31, 2012, using the Hospital Episode Statistics in England, and the Swedish Medical Birth Register in Sweden, with longitudinal follow-up from linked hospital admissions and mortality records. We analysed mortality as the outcome, based on deaths from any cause at age 2–27 days, 28–364 days, and 1–4 years. We fitted Cox proportional hazard regression models to estimate the hazard ratios (HRs) for England compared with Sweden in all three age groups. The models were adjusted for birth characteristics (gestational age, birthweight, sex, and congenital anomalies), and for socioeconomic factors (maternal age and socioeconomic status).

**Findings:**

The English cohort comprised 3 932 886 births and 11 392 deaths and the Swedish cohort comprised 1 013 360 births and 1927 deaths. The unadjusted HRs for England compared with Sweden were 1·66 (95% CI 1·53–1·81) at 2–27 days, 1·59 (1·47–1·71) at 28–364 days, and 1·27 (1·15–1·40) at 1–4 years. At 2–27 days, 77% of the excess risk of death in England was explained by birth characteristics and a further 3% by socioeconomic factors. At 28–364 days, 68% of the excess risk of death in England was explained by birth characteristics and a further 11% by socioeconomic factors. At 1–4 years, the adjusted HR did not indicate a significant difference between countries.

**Interpretation:**

Excess child mortality in England compared with Sweden was largely explained by the unfavourable distribution of birth characteristics in England. Socioeconomic factors contributed to these differences through associations with adverse birth characteristics and increased mortality after 1 month of age. Policies to reduce child mortality in England could have most impact by reducing adverse birth characteristics through improving the health of women before and during pregnancy and reducing socioeconomic disadvantage.

**Funding:**

The Farr Institute of Health Informatics Research (through the Medical Research Council, Arthritis Research UK, British Heart Foundation, Cancer Research UK, Chief Scientist Office, Economic and Social Research Council, Engineering and Physical Sciences Research Council, National Institute for Health Research, National Institute for Social Care and Health Research, and the Wellcome Trust).

## Introduction

The UK has one of the highest child mortality rates in western Europe. In 2013, mortality in children aged less than 5 years was 4·9 per 1000 births in the UK, which was around 25% higher compared with France, Germany, Italy, or Spain, and almost twice as high compared with Sweden (2·7 per 1000 births).^1^ As both the UK and Sweden have comparable standards of economic development and universal health care, Sweden is often viewed as a benchmark for reductions in child mortality that should be achievable in the UK.^2^, ^3^, ^4^, ^5^

Policy makers and child health professionals have called for improvements in child health services to reduce child mortality in the UK relative to Sweden, including more efficient general practitioner (GP) responses to sick children through better integration of GP and paediatric services and enhanced training.^2^, ^4^, ^5^ However, the higher rate of child mortality in the UK could also reflect a higher prevalence of risk factors at birth, such as prematurity, low birthweight, and congenital anomalies,^2^, ^6^, ^7^ as over 80% of deaths in children under 5 years occur before 12 months (86% in the UK and 81% in Sweden in 2013).^1^ An unequal distribution of wealth in the UK's society has also been reported to contribute to the high observed child mortality.^2^, ^5^, ^7^, ^8^, ^9^

Policy makers need evidence from a comparison of child mortality between the UK and Sweden that accounts for between-country differences in birth characteristics (including birthweight, gestational age, and congenital anomalies) and socioeconomic circumstances to guide policies to reduce child mortality. Such evidence could inform policy decisions on whether to focus preventive strategies on health care for children after birth or on improving the health of women before and during pregnancy to reduce risk factors present at birth.

We compared child mortality in England, which accounts for 85% of all births in the UK,^10^ and Sweden using individual-level, national administrative health databases. We aimed to identify how much of the increase in child mortality in England compared with Sweden is explained by differences in prevalence of adverse birth characteristics and socioeconomic factors operating after birth.

**Research in context**

**Evidence before the study**

We searched PubMed for international comparisons of child mortality published from Jan 1, 2000, to Jun 30, 2017, which included England or the UK and Sweden in the analyses. We used the search terms “((England OR English OR United Kingdom OR UK) AND (Sweden OR Swedish))” AND “(infan* OR neonat* OR post-neonat* OR child OR childhood OR under-5)” AND “(death OR mortality OR dying OR survival)” in titles and abstracts, and checked reference lists of identified papers to find additional relevant studies. We excluded studies which only used data from before the year 2000, and included only studies in English. We identified seven studies. Global comparisons of trends in child mortality show that rates in the UK have remained almost twice those in Sweden. Potential explanations are the UK's higher prevalence of preterm birth and congenital anomalies and that the UK has one of the most unequal distributions of income of all western countries. In 2003–05, the most deprived 20% of the population had a seven-times lower income than did the least deprived 20%, compared with a four-times difference in Sweden. Additionally, differences in health-care provision might contribute to increased health-care amenable mortality in the UK relative to Sweden. However, no previous study has adequate data to identify the relative contribution of birth characteristics or socioeconomic status to the excess in child mortality in the UK compared with Sweden.

**Added value of this study**

We developed representative birth cohorts in England and Sweden using individual-level administrative health databases with detailed information on child characteristics at birth (birthweight, gestational age, congenital anomalies, and sex) and socioeconomic factors (maternal age and socioeconomic status). We compared mortality in England and Sweden adjusted for these risk factors at birth. At 2–27 days, 77% of the excess mortality in England relative to Sweden was due to differences in birth characteristics and 3% due to an independent effect of socioeconomic factors. For mortality at 28–364 days, these proportions were 68% for birth characteristics and 11% for socioeconomic factors. Small but significant differences remained after adjustment in the first year of life. At 1–4 years, the differences in mortality were negligible after adjusting for birth characteristics and socioeconomic factors.

**Implications of all the available evidence**

The largest improvements in child mortality in England relative to Sweden could be achieved by reducing the prevalence of low birthweight, prematurity, and congenital anomalies. The risk of child mortality in England relative to Sweden declined by 70–80% when adjusted for birth characteristics in the two populations. Socioeconomic disadvantage contributes to child mortality through its association with adverse birth characteristics, and in our study socioeconomic factors independently led to a further 10% reduction in mortality beyond the first month of life. These findings suggest that the biggest reductions in child mortality in England relative to Sweden could be achieved by reducing the prevalence of adverse birth characteristics through universal programmes to improve the health of women and reduce health inequalities before and during pregnancy.

## Methods

### Study design and participants

In this population-based birth cohort study, we developed nationally representative birth cohorts using birth records linked to hospital admission data and mortality registration databases in England and Sweden. These databases captured 97% of all births and 98–99% of all hospital admissions in England,^11^ and 98–99% of births^12^ and all hospital admissions in Sweden.^13^ The cohorts comprised singleton livebirths between Jan 1, 2003, and Dec 31, 2012, to resident mothers in each country. We followed up children until their fifth birthday, death, or Dec 31, 2013, whichever occurred first.

We received ethical approval to use the Swedish national registers from the Regional Committee of Stockholm (no 2016/1234-31/5; approved on Aug 4, 2016). We have a data sharing agreement with National Health Service (NHS) Digital to use a de-identified extract of Hospital Episode Statistics linked to Office for National Statistics death registration data for research on child mortality; therefore, we did not require ethical approval to use English datasets.^14^

In England, entry to the birth cohort was based on birth admissions recorded in Hospital Episode Statistics, which is a hospital discharge database containing all admissions to NHS hospitals and 97% of all births in England.^11^ Hospital Episode Statistics birth records include child and maternal characteristics (eg, birthweight, gestational age, and maternal age). We excluded hospitals with high proportions of missing data or evidence of linkage error to address incomplete recording of risk factors at birth. We included hospitals with more than 500 births a year, with high completeness of recorded birthweight and gestational age, and hostpitals where at least half of all deaths were linked to a death certificate (see appendix for selection criteria). We ensured representativeness of the cohort by comparing distributions of birthweight, gestational age, and maternal age among livebirths and infant deaths against national statistics for England and Wales published by the Office for National Statistics (appendix).^15^, ^16^ We followed up the cohort by linking each child's birth admission with subsequent hospital admissions and Office for National Statistics death registrations. NHS Digital generated a study-specific identifier (called the HESID) to enable us to link hospital records across time and with mortality records using the patient's NHS number, postcode, date of birth, sex, and hospital patient identifier.^17^

In Sweden, entry to the birth cohort was based on data recording in the Swedish Medical Birth Register, a mandatory database containing details of antenatal, obstetric, and neonatal care for all births in Sweden to resident mothers.^12^ Singleton livebirths recorded in the Swedish Medical Birth Register were linked to the Swedish Hospital Discharge Register^13^ and the Swedish Cause of Death Register^18^ through each child's study-specific identifier based on their unique personal identity number.^19^

### Risk factors

Information about birthweight, gestational age, and sex was obtained from the Swedish Medical Birth Register and from Hospital Episode Statistics.^11^, ^12^ In the English cohort, we linked the baby's birth admissions with maternal delivery admissions within Hospital Episode Statistics to minimise missing values of birth characteristics and socioeconomic factors (appendix).^20^ The Swedish Medical Birth Register had near complete recording of the risk factors in this study.

We categorised gestational age as 24–27 weeks, 28–31 weeks, 32–34 weeks, 35–36 weeks, 37–38 weeks, and 39 weeks and longer, and birthweight as 500–999 g, 1000–1499 g, 1500–2499 g, 2500–3499 g, and 3500 g and higher. To minimise bias from intercountry differences in recording of stillbirths and early neonatal deaths, we excluded all babies weighing less than 500 g at birth or born at less than 24 weeks of gestation in both England and Sweden.^21^, ^22^ We categorised children as having a congenital anomaly if they had any relevant International Classification of Diseases version 10 codes recorded at birth, during any admission before 2 years of age, or on a death certificate as any cause of death, using a subgroup of codes from a list of chronic conditions in children.^23^

No directly comparable measure of socioeconomic status was available in the two countries. Instead, in Sweden we used quintiles of disposable household income in the year before childbirth, obtained via linkage to the Longitudinal Integration Database for Health Insurance and Labour Market Studies.^24^ In England, we used quintiles of the Index of Multiple Deprivation score, a small area indicator measured per 200–1400 households.^11^ Index of Multiple Deprivation scores were allocated using the child's postcode recorded at birth (on admission of the baby or mother), or in hospital admissions during infancy.

As young maternal age (<20 years) is strongly associated with social disadvantage^25^ it can be used as an additional comparable indicator of socioeconomic status. Maternal age was recorded in the Swedish Medical Birth Register and in birth and delivery admission records in Hospital Episode Statistics, and was categorised as less than 20 years, 20–24 years, 25–29 years, 30–34 years, 35–39 years, and 40 years and older.

### Outcomes

We analysed mortality as the outcome, based on deaths from any cause at age 2–27 days, 28–364 days, and 1–4 years. We excluded deaths on days 0–1 because of under-reporting of risk factors at birth for these deaths in the English cohort (appendix) and to minimise bias from intercountry differences in recording of stillbirths and early neonatal deaths.

### Statistical analysis

We derived the numbers and proportions of livebirths and deaths according to each birth characteristic (birthweight, gestational age, sex, and congential anomalies) and socioeconomic factor (maternal age and socioeconomic status) in England and Sweden. We calculated unadjusted mortality rates per 100 000 child-years by country and each risk factor category. We plotted Kaplan-Meier failure curves to illustrate between-country differences in the distribution of deaths by age, according to gestational age and presence of congenital anomalies. For each plot, we calculated the number of excess deaths in England relative to Sweden by multiplying the number of singleton livebirths in all of England (before applying exclusion criteria) between Jan 1, 2003, and Dec 31, 2012 (n=6 100 404), by the difference in the proportion of children who died in England and Sweden (by their first and fifth birthdays). We also listed the ten most common congenital anomalies recorded in hospital records and death certificates in each country.

We fitted Cox proportional hazards regression models to estimate hazard ratios (HRs), comparing mortality in England relative to Sweden (the baseline). We fitted separate models for deaths at 2–27 days, 28–364 days, and 1–4 years. We first fitted unadjusted models including only country of birth. We then added birth characteristics and socioeconomic factors a priori. To estimate the extent to which the differences in birth characteristics and socioeconomic factors contributed to the increased risk of death in England relative to Sweden, we calculated the percentage of excess risk mediated (abbreviated as PERM)^26^, ^27^ and split it into two components as follows:

$$\text{PERM}={\text{PERM}}_{\text{birth}\;\text{characteristics}}+{\text{PERM}}_{\text{socioeconomic}\;\text{factors}}$$

where

$$\frac{\text{HR}(\text{unadjusted})-\text{HR}(\text{adjusted}\;\text{for}\;\text{birth}\;\text{characteristics})}{HR(\text{unadjusted})-1}\times 100$$

and

$${\text{PERM}}_{\text{socioeconomic}\;\text{factors}}=\frac{\text{HR}(\text{adjusted}\;\text{for}\;\text{birth}\;\text{characteristics})-\text{HR}(\text{adjusted}\;\text{for}\;\text{birth}\;\text{characteristics and}\;\text{socioeconomic}\;\text{factors})}{\text{HR}(\text{unadjusted})-1}\times 100$$

Percentage of excess risk mediated was calculated only for models with a statistically significant adjusted HR for England compared with Sweden, when the Wald test result for the country parameter was p<0·05. For all models, the proportional hazards assumption was assessed using Schoenfeld residual plots.^28^

As England and Sweden might differ in the recording of congenital anomalies, we used a stricter definition, indicating only severe congenital anomalies in sensitivity analyses.^29^ We also fitted additional Cox proportional hazards regression models, including an effect modification term with age for congenital anomaly, for models where the proportional hazards assumption was not met. We used Stata MP version 14.2 for all analyses.

### Role of the funding source

The funders of the study had no role in study design, data collection, data analysis, data interpretation, or writing of the report. The corresponding author had full access to all the data in the study and had final responsibility for the decision to submit for publication.

## Results

**Figure 1:**
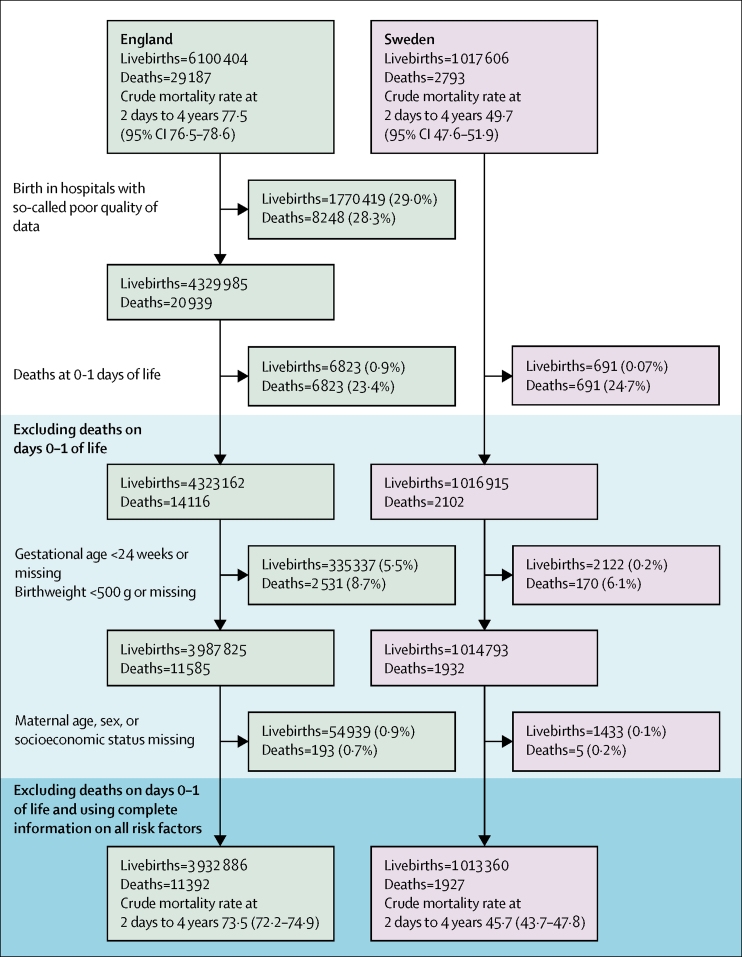
Development of comparable and representative birth cohorts in England and Sweden For each exclusion criterion, the percentages of all livebirths and all deaths are shown in brackets. Crude mortality rates at age 2 days to 4 years per 100 000 person-years are presented for each country before and after applying all exclusion criteria.

The English cohort comprised 3 932 886 births and 11 392 deaths and the Swedish cohort comprised 1 013 360 births and 1927 deaths (figure 1). This represented 64·5% of all singleton livebirths and 58·4% of all deaths in children aged 2 days to 4 years in England, and 99·8% of all singleton livebirths and 91·7% of all deaths in children aged 2 days to 4 years in Sweden.

In general, children born in England weighed less at birth and were born at earlier gestational ages than were children born in Sweden (table 1). In England, more children were born with congenital anomalies compared with Sweden, but the types of anomalies recorded were similar in the two countries (appendix). There were four times more mothers aged younger than 20 years in England compared with Sweden.

**Table 1:** Sociodemographic characteristics of singleton livebirths and children who died in England and Sweden

		**Livebirths**		**Deaths at 2–27 days**		**Deaths at 28–364 days**		**Deaths at 1–4 years**	
		England	Sweden	England	Sweden	England	Sweden	England	Sweden
Total		3 932 886	1 013 360	4207	648	4964	803	2223	476
Birthweight, g									
	500–999	9458 (0·2%)	1742 (0·2%)	1067 (25·4%)	129 (19·9%)	651 (13·1%)	76 (9·5%)	55 (2·5%)	4 (0·8%)
	1000–1499	18 288 (0·5%)	3102 (0·3%)	391 (9·3%)	63 (9·7%)	279 (5·6%)	42 (5·2%)	52 (2·3%)	10 (2·1%)
	1500–2499	190 299 (4·8%)	25 817 (2·5%)	774 (18·4%)	133 (20·5%)	962 (19·4%)	136 (16·9%)	306 (13·8%)	47 (9·9%)
	2500–3499	2 090 583 (53·2%)	429 107 (42·3%)	1425 (33·9%)	203 (31·3%)	2261 (45·5%)	342 (42·6%)	1217 (54·7%)	216 (45·4%)
	≥3500	1 624 258 (41·3%)	553 592 (54·6%)	550 (13·1%)	120 (18·5%)	811 (16·3%)	207 (25·8%)	593 (26·7%)	199 (41·8%)
Gestational age, weeks									
	24–27	8806 (0·2%)	1769 (0·2%)	1043 (24·8%)	135 (20·8%)	604 (12·2%)	78 (9·7%)	52 (2·3%)	3 (0·6%)
	28–31	22 327 (0·6%)	4354 (0·4%)	442 (10·5%)	64 (9·9%)	314 (6·3%)	39 (4·9%)	46 (2·1%)	7 (1·5%)
	32–34	56 093 (1·4%)	11 764 (1·2%)	318 (7·6%)	63 (9·7%)	320 (6·4%)	50 (6·2%)	71 (3·2%)	24 (5·0%)
	35–36	137 046 (3·5%)	30 295 (3·0%)	351 (8·3%)	71 (11·0%)	447 (9·0%)	81 (10·1%)	144 (6·5%)	28 (5·9%)
	37–38	726 907 (18·5%)	191 130 (18·9%)	707 (16·8%)	117 (18·1%)	1127 (22·7%)	190 (23·7%)	516 (23·2%)	101 (21·2%)
	≥39	2 981 707 (75·8%)	774 048 (76·4%)	1346 (32·0%)	198 (30·6%)	2152 (43·4%)	365 (45·5%)	1394 (62·7%)	313 (65·8%)
Sex									
	Male	2 016 683 (51·3%)	520 985 (51·4%)	2388 (56·8%)	368 (56·8%)	2828 (57·0%)	457 (56·9%)	1202 (54·1%)	260 (54·6%)
	Female	1 916 203 (48·7%)	492 375 (48·6%)	1819 (43·2%)	280 (43·2%)	2136 (43·0%)	346 (43·1%)	1021 (45·9%)	216 (45·4%)
Congenital anomaly									
	No	3 817 789 (97·1%)	988 681 (97·6%)	2376 (56·5%)	365 (56·3%)	2724 (54·9%)	473 (58·9%)	1386 (62·3%)	358 (75·2%)
	Yes	115 097 (2·9%)	24 679 (2·4%)	1831 (43·5%)	283 (43·7%)	2240 (45·1%)	330 (41·1%)	837 (37·7%)	118 (24·8%)
Maternal age, years									
	<20	241 503 (6·1%)	16 160 (1·6%)	373 (8·9%)	11 (1·7%)	571 (11·5%)	34 (4·2%)	189 (8·5%)	16 (3·4%)
	20–24	758 596 (19·3%)	129 240 (12·8%)	888 (21·1%)	102 (15·7%)	1193 (24·0%)	153 (19·1%)	553 (24·9%)	73 (15·3%)
	25–29	1 064 469 (27·1%)	295 905 (29·2%)	1131 (26·9%)	163 (25·2%)	1254 (25·3%)	233 (29·0%)	571 (25·7%)	131 (27·5%)
	30–34	1 110 202 (28·2%)	356 356 (35·2%)	977 (23·2%)	222 (34·3%)	1114 (22·4%)	218 (27·1%)	555 (25·0%)	156 (32·8%)
	35–39	617 394 (15·7%)	178 992 (17·7%)	635 (15·1%)	108 (16·7%)	629 (12·7%)	126 (15·7%)	287 (12·9%)	80 (16·8%)
	≥40	140 722 (3·6%)	36 707 (3·6%)	203 (4·8%)	42 (6·5%)	203 (4·1%)	39 (4·9%)	68 (3·1%)	20 (4·2%)
Quintile of socioeconomic status									
	Q1	852 422 (21·7%)	201 613 (19·9%)	1248 (29·7%)	166 (25·6%)	1589 (32·0%)	251 (31·3%)	627 (28·2%)	115 (24·2%)
	Q2	804 432 (20·5%)	200 440 (19·8%)	954 (22·7%)	129 (19·9%)	1228 (24·7%)	168 (20·9%)	489 (22·0%)	114 (23·9%)
	Q3	768 484 (19·5%)	202 670 (20·0%)	777 (18·5%)	88 (13·6%)	842 (17·0%)	107 (13·3%)	422 (19·0%)	94 (19·7%)
	Q4	763 076 (19·4%)	204 215 (20·2%)	662 (15·7%)	93 (14·4%)	736 (14·8%)	132 (16·4%)	366 (16·5%)	85 (17·9%)
	Q5	744 472 (18·9%)	204 422 (20·2%)	566 (13·5%)	172 (26·5%)	569 (11·5%)	145 (18·1%)	319 (14·3%)	68 (14·3%)

Q1 denotes the most deprived 20% of children. Q5 denotes the least deprived 20% of children. Q=quintile.

**Figure 2:**
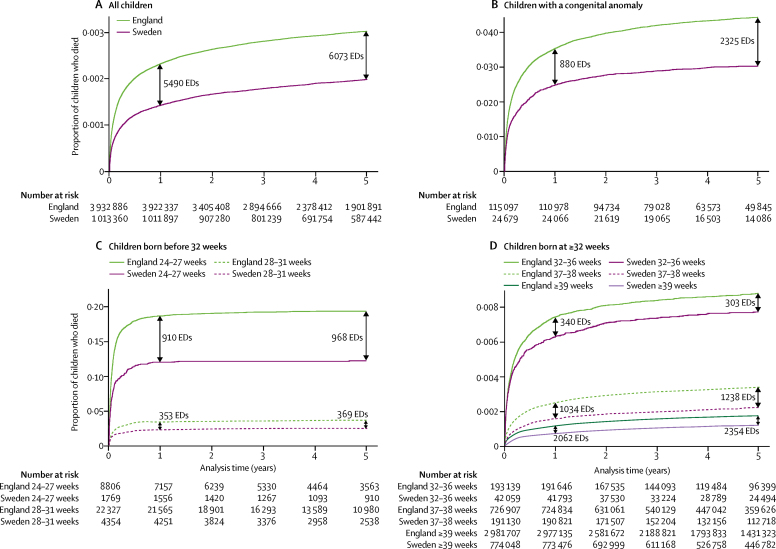
Mortality at 2 days to 4 years in England and Sweden, overall and by selected risk factors at birth We calculated excess deaths at 1 year and 5 years by multiplying the number of births in the English cohort based on all births (n=6 100 404) by the difference in proportion of children who died by their first or fifth birthday in England and Sweden. Probability of death is only presented for one risk factor at a time. ED=excess death.

The unadjusted child mortality rate was almost twice as high in England compared with Sweden (table 2), accounting for about 607 excess deaths per year at 2 days to 4 years of age in England (6073 in total in 2003–12; figure 2). Unadjusted mortality rates were highest for high risk groups (eg, gestational age 24–27 weeks, birthweight 500–1499 g, and those with congenital anomalies; table 2), but absolute numbers of excess deaths in England were largest for low risk but high prevalence birth characteristics, such as birth at full term, and for children born with congenital anomalies (figure 2).

**Table 2:** Unadjusted mortality rates overall and by risk factors at birth in England and Sweden (univariate analyses)

		**England**				**Sweden**			
		2–27 days	28–364 days	1–4 years	2 days–4 years	2–27 days	28–364 days	1–4 years	2 days–4 years
Overall		1500 (1500–1600)	140 (130–140)	19 (18–20)	74 (72–75)	920 (850–990)	86 (80–92)	15 (13–16)	46 (44–48)
Birthweight, g									
	500–999	180 000 (170 000–190 000)	9000 (8300–9700)	230 (180–310)	5700 (5500–6000)	110 000 (94 000–130 000)	5300 (4200–6600)	81 (30–220)	3200 (2800–3700)
	1000–1499	31 000 (28 000–35 000)	1700 (1500–1900)	98 (75–130)	1000 (950–1100)	30 000 (23 000–38 000)	1500 (1100–2100)	93 (49–180)	910 (760–1100)
	1500–2499	5800 (5400–6300)	550 (520–590)	54 (48–61)	270 (260–280)	7400 (6200–8800)	570 (490–680)	58 (44–77)	300 (270–330)
	2500–3499	980 (930–1000)	120 (110–120)	20 (19–21)	59 (58–61)	680 (590–780)	87 (78–96)	16 (14–18)	43 (40–46)
	≥3500	490 (450–530)	54 (50–58)	12 (12–14)	31 (29–32)	310 (260–370)	41 (35–47)	11 (9·8–13)	23 (21–25)
Gestational age, weeks									
	24–27	190 000 (170 000–200 000)	9000 (8300–9800)	240 (180–320)	6000 (5700–6200)	120 000 (98 000–140 000)	5400 (4300–6700)	60 (19–190)	3300 (2900–3700)
	28–31	29 000 (26 000–32 000)	1600 (1400–1700)	71 (53–95)	930 (870–1000)	21 000 (17000–27 000)	990 (720–1400)	52 (25–110)	620 (510–740)
	32–34	8200 (7300–9100)	620 (560–690)	43 (34–54)	320 (300–340)	7700 (6000–9900)	470 (360–620)	62 (41–93)	280 (240–330)
	35–36	3600 (3300–4000)	350 (320–380)	35 (30–41)	170 (160–180)	3400 (2700–4200)	290 (230–360)	29 (20–42)	140 (120–170)
	37–38	1400 (1300–1500)	170 (160–180)	24 (22–26)	82 (78–85)	880 (730–1100)	110 (93–120)	17 (14–20)	51 (46–56)
	≥39	650 (610–680)	78 (75–82)	16 (15–17)	42 (41–43)	370 (320–420)	51 (46–57)	13 (11–14)	27 (26–29)
Sex									
	Male	1700 (1600–1800)	150 (150–160)	20 (19–21)	81 (79–83)	1000 (910–1100)	96 (87–100)	16 (14–18)	50 (47–53)
	Female	1400 (1300–1400)	120 (120–130)	18 (17–19)	66 (64–68)	810 (720–920)	76 (69–85)	14 (12–16)	41 (38–44)
Congenital anomaly									
	No	890 (860–930)	77 (74–80)	12 (12–13)	43 (42–44)	530 (480–590)	52 (48–57)	11 (10–13)	29 (27–31)
	Yes	23 000 (22 000–24 000)	2200 (2100–2300)	260 (250–280)	1100 (1100–1200)	17 000 (15 000–19 000)	1500 (1300–1600)	150 (130–190)	730 (680–780)
Maternal age, years									
	<20	2200 (2000–2400)	260 (240–280)	25 (22–29)	110 (110–120)	980 (540–1800)	230 (160–320)	31 (19–50)	89 (70–110)
	20–25	1700 (1600–1800)	170 (160–180)	25 (23–27)	88 (85–91)	1100 (930–1400)	130 (110–150)	18 (14–23)	62 (55–69)
	25–30	1500 (1400–1600)	130 (120–130)	18 (17–20)	71 (69–74)	790 (680–920)	86 (75–97)	14 (12–16)	43 (39–47)
	30–35	1300 (1200–1300)	110 (100–120)	17 (16–19)	61 (59–63)	890 (780–1000)	67 (59–77)	13 (11–16)	40 (37–43)
	35–40	1500 (1400–1600)	110 (100–120)	16 (14–18)	63 (60–66)	860 (720–1000)	76 (64–91)	14 (11–18)	43 (38–47)
	≥40	2100 (1800–2400)	160 (140–180)	17 (13–22)	87 (80–95)	1600 (1200–2200)	120 (84–160)	18 (11–27)	68 (56–82)
Quintile of socioeconomic status									
	Q1	210 (200–220)	20 (19–21)	2·5 (2·3–2·7)	10 (9·9–11)	120 (100–140)	14 (12–15)	1·8 (1·5–2·1)	6·4 (5·9–6·9)
	Q2	170 (160–180)	17 (16–17)	2·1 (1·9–2·2)	8·5 (8·2–8·8)	92 (78–110)	9·1 (7·8–11)	1·8 (1·5–2·1)	4·9 (4·5–5·4)
	Q3	140 (130–150)	12 (11–13)	1·8 (1·7–2·0)	6·7 (6·4–7·0)	62 (50–77)	5·7 (4·7–6·9)	1·5 (1·2–1·8)	3·4 (3·0–3·8)
	Q4	120 (120–130)	10 (9·7–11)	1·7 (1·5–1·9)	5·9 (5·7–6·2)	65 (53–80)	7·0 (5·9–8·3)	1·3 (1·0–1·6)	3·6 (3·3–4·1)
	Q5	110 (100–120)	8·3 (7·6–9·0)	1·4 (1·3–1·6)	5·0 (4·7–5·2)	120 (100–140)	7·7 (6·5–9·0)	1·1 (0·83–1·3)	4·5 (4·1–5·0)

Data are unadjusted mortality rates per 100 000 child-years (95% CI). Q1 denotes the most deprived 20% of children. Q5 denotes the least deprived 20% of children. Q=quintile.

**Figure 3:**
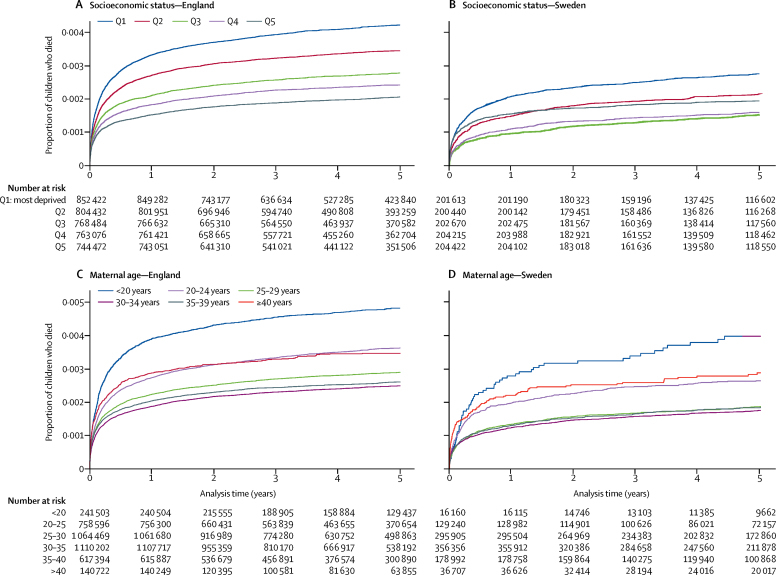
Survival at 2 days to 4 years by socioeconomic factors in England and in Sweden Q1 denotes the most deprived 20% of pregnant women. Q5 represents the least deprived 20% of pregnant women. Probability of death is only presented for one risk factor at a time. Q=quintile.

Disparities in child mortality by socioeconomic status were wider in England than in Sweden (figure 3); however, the area-level socioeconomic status indicator in England and individual-level socioeconomic status indicator in Sweden were not directly comparable. We found significant between-country differences for all levels of socioeconomic status, apart from the least deprived 20% of children (table 2). Mortality rates by maternal age showed similar patterns in the two countries (figure 3). However, mortality rates were higher in England than in Sweden for all maternal age groups (table 2).

At 2–27 days of age, the unadjusted HR for England relative to Sweden was 1·66 (95% CI 1·53–1·81; table 3). After adjustment for birth characteristics, the HR for country decreased to 1·15 (1·06–1·25), and further adjustment for socioeconomic factors reduced the HR to 1·13 (1·04–1·23). Between-country differences in the distribution of birth characteristics explained 77% of the excess risk of death in England relative to Sweden. A further 3% was explained by socioeconomic factors, over and above their effect on birth characteristics. In the fully adjusted model (for birth characteristics and socioeconomic factors), birthweight less than 1500 g, gestational age less than 28 weeks, and congenital anomalies were associated with the highest risk of death at 2–27 days (appendix).

**Table 3:** Differences in child mortality between England and Sweden attributable to birth characteristics and socioeconomic factors

	**2–27 days**		**28–364 days**		**1–4 years**	
	HR (95% CI)	Percentage excess risk mediated	HR (95% CI)	Percentage excess risk mediated	HR (95% CI)	Percentage excess risk mediated
Unadjusted model*	1·66 (1·53–1·81)	..	1·59 (1·47–1·71)	..	1·27 (1·15–1·40)	..
Model adjusted for birth characteristics†	1·15 (1·06–1·25)	77%	1·19 (1·10–1·28)	68%	1·10 (1·00–1·22)	NA
Model adjusted birth characteristics and socioeconomic factors‡	1·13 (1·04–1·23)	3%	1·12 (1·04–1·21)	11%	1·06 (0·96–1·18)	NA

HR=hazard ratio for England relative to Sweden (baseline). NA=not applicable.

* Cox proportional hazards regression model adjusted only for country.

† Cox proportional hazards regression model adjusted for country, birthweight, gestational age, sex, and congenital anomalies.

‡ Cox proportional hazards regression model adjusted for country, birthweight, gestational age, sex, congenital anomalies, maternal age, and socioeconomic status.

At 28–364 days, the unadjusted HR for England relative to Sweden was 1·59 (95% CI 1·47–1·71; table 3). The HR decreased to 1·19 (1·10–1·28) after adjustment for birth characteristics, and to 1·12 (1·04–1·21) after adjustment for socioeconomic factors. Between-country differences in the distribution of birth characteristics accounted for 68% of the excess risk of death in England relative to Sweden, and socioeconomic factors independently explained a further 11%. In the fully adjusted model, the highest risk of death at 28–364 days was associated with the presence of a congenital anomaly and birthweight less than 1500 g (appendix).

At 1–4 years, the unadjusted HR for England relative to Sweden was 1·27 (95% CI 1·15–1·40; table 3). The HR dropped to 1·10 (1·00–1·22) after adjustment for birth characteristics. After further adjustment for socioeconomic factors, there were no significant differences in child mortality between England and Sweden (1·06, 0·96–1·18). Presence of a congenital anomaly was the single most important risk factor for death at 1–4 years in the fully adjusted model, increasing the risk of death by 17 times, followed by low birthweight (<2500 g) with a four times increase (appendix).

The results did not change appreciably in sensitivity analyses using an indicator for severe congenital anomaly (appendix), or when we included an effect modification term with age for congenital anomaly to meet the proportional hazard assumption (appendix).

## Discussion

Child mortality was substantially higher at all ages in England compared with Sweden; however, birth characteristics largely explained the increased risk of death. Differences in the distribution of birth characteristics accounted for 77% of the excess risk of death in England at 2–27 days and 68% at 28–364 days. Socioeconomic factors accounted for a further 3% of the increased risk of death in England relative to Sweden at 2–27 days and 11% at 28–364 days, independent of birth characteristics. A small but significantly increased risk of death in the first year of life in England remained after adjustment for birth characteristics and socioeconomic factors. However, differences in mortality beyond the first year of life were negligible in the fully adjusted model.

A strength of our study was the use of nationally representative birth cohorts, with detailed birth characteristics and linkage to all hospital admission records and death registrations up to 4 completed years of age. Child deaths are rare, but the large sample of individual-level data enabled us to investigate associations for low prevalence risk factors, such as congenital anomalies and extreme prematurity, both alone and in combination. Our results were robust to all sensitivity analyses. Our approach to inter-country comparisons overcomes the limitations of using aggregate data and could serve as a model for future international comparisons of child mortality and other health outcomes in countries with electronic health records.

A limitation of this study is the quality of data in England. We excluded a third of births because of incomplete recording of birth characteristics in the hospital admission database. We therefore based our analyses on a representative subgroup of births in England, validated against national statistics. A national birth cohort with almost 100% completeness of risk factors at birth and high quality of linkage to mortality data could be developed by linking Office for National Statistics birth registration, NHS birth notification data, and Hospital Episode Statistics records for mothers and babies in England. The feasibility of such linkage has been shown previously.^30^, ^31^

Another limitation of our study is that we excluded deaths occurring on days 0 and 1 of life because of missing data on risk factors in England and to minimise bias due to inter-country differences in classification of stillbirths and livebirths. Deaths on days 0 and 1 of life accounted for around a quarter of deaths in children aged less than 5 years in England and Sweden. Adverse birth characteristics are key determinants of mortality in the first week of life,^32^ and we showed that their prevalence is higher in England than in Sweden. Therefore, our results probably underestimate the contribution of adverse birth characteristics to the increased risk of neonatal mortality in England compared with Sweden. Further comparisons, including both stillbirths and livebirths (to allow for between-country differences in definitions and mortality registration practices), are needed to confirm this hypothesis. Comparisons based on all livebirths and stillbirths would also minimise the so-called livebirth bias, which arises when the same prenatal exposures are associated with the outcome of interest (here child mortality) and the risk of fetal death.^33^

A third limitation of our study is the use of different measures for socioeconomic status in England (an area-level indicator) and Sweden (an individual-level indicator of household income). Maternal education is the most comparable and consistent socioeconomic status indicator for international studies of child health outcomes,^34^, ^35^ but such data are not available in England. Furthermore, we only accounted for the effect of socioeconomic factors on excess child mortality in England relative to Sweden, independent of birth characteristics. Further comparisons using causal mediation methods are needed to establish the total contribution of socioeconomic factors to increased child mortality in England relative to Sweden (ie, including both the effect mediated by low birthweight, preterm birth, and congenital anomalies, and the direct effect).

Our study would have benefited from analysis of additional maternal risk factors, such as smoking during pregnancy and body-mass index (BMI), which are recorded in the Swedish Medical Birth Register,^12^ but not in any national birth or maternity datasets in England. We could not establish the contribution of ethnicity to the differences in child mortality between England and Sweden. Ethnic group data are collected in England using a UK-specific categorisation, and comparable ethnic group information is not collected in Sweden. However, the mother's country of birth is recorded both in the Swedish Medical Birth Register^12^ and Office for National Statistics birth registration.^31^ Further work could explore differences in infant and child mortality according to the immigration history of mothers in the different health and social policy contexts of England and Sweden.

Disparities in child mortality rates in England compared with Sweden were largely driven by differences in distribution of birth characteristics in the two countries. A child's birth characteristics are associated with maternal characteristics, health, and socioeconomic circumstances before and during pregnancy. For example, young or old maternal age, smoking during pregnancy, and obesity are associated with increased prevalence of preterm birth,^36^, ^37^, ^38^ intrauterine growth restriction,^37^, ^38^ and some congenital anomalies.^39^ Prevalence of many of these factors is higher in England than in Sweden. For example, prevalence of smoking during pregnancy is almost twice as high in England as in Sweden (12% *vs* 6·5% in 2010), and a higher proportion of women are obese (in 2010, one in five women in England had a BMI ≥30 kg/m^2^ *vs* one in eight in Sweden).^32^, ^40^ Therefore, policies to reduce child mortality in England need to focus on improving maternal health before and during pregnancy.

Efforts to reduce the prevalence of adverse birth characteristics would not only reduce child mortality, but could improve many other serious and more prevalent health problems. Preterm birth and low birthweight are associated with increased risk of chronic illness (such as cerebral palsy or sensory impairment),^41^, ^42^, ^43^, ^44^ respiratory illness,^43^ and mental health problems.^45^ Growing evidence for associations with low educational attainment^41^, ^42^, ^43^ and increased risk of long-term adult health outcomes, including cardiovascular disease, hypertension, and diabetes, has been found.^43^, ^46^ Our findings support recommendations made by others^46^, ^47^ that the benefits from investing limited public resources to improve maternal health before and during pregnancy have lifelong health benefits for a substantial proportion of the population, far exceeding the number of children who stand to benefit by reducing child mortality.

Further research needs to focus on differences in the prevalence of adverse birth characteristics within each country according to maternal health indicators (eg, obesity, infections, or presence of chronic conditions such as diabetes or hypertensive disorders), health behaviours (smoking, drug or alcohol use during pregnancy, and nutrition), ethnic group, and measures of health inequalities and socioeconomic disadvantage of the mother to evaluate policy interventions to improve maternal health and reduce adverse birth characteristics. Welfare policies that reduce socioeconomic disparities in England should be an important part of a strategy to lower child mortality in England. Socioeconomic disadvantage is an important determinant of preterm birth,^48^, ^49^ low birthweight,^48^, ^49^ presence of congenital anomalies,^50^ and associated maternal risk factors, operating upstream through the responses and behaviours of mothers exposed to poverty, stress, and financial hardship.^48^ The UK has a higher proportion of children living in relative poverty (12·1% *vs* 7·3% in Sweden in 2009) or in deprived households (5·5% *vs* 1·3% in Sweden in 2009).^51^ Our study also found that teenage mothers, who are more likely to come from deprived backgrounds,^25^ accounted for four times more births in England than in Sweden. Therefore, the increased prevalence of adverse birth characteristics in England could at least partly be attributed to a larger population being exposed to socioeconomic disadvantage.

Some of the increased prevalence of congenital anomalies in England might be attributable to differences in provision of prenatal care. For example, the number of terminations of pregnancies is higher in Sweden than in England because of more terminations for women aged 35 years and older (numbers are comparable for women aged <20 years)^52^ and screening for chromosomal anomalies.^53^ These differences could reflect differing cultural attitudes to termination of pregnancy, in timing of detection of the anomaly (ultrasound scan is offered at 15–18 weeks in Sweden compared with 18–21 weeks in England),^54^ or in uptake of the scan; in 2016, more than 90% of women attended antenatal care before 20 weeks of pregnancy in both countries, but more than 95% of women in Sweden had an ultrasound scan compared with an estimated 75–95% in England and Wales.^55^

Further research is needed to identify the origins of the small but significant differences in the risk of death, which remained after adjustment for characteristics at birth and socioeconomic factors. First, some of these differences could reflect variation in the prevalence or severity of chronic conditions not measured in our study that are associated with increased mortality (such as cerebral palsy). Second, differences between England and Sweden might reflect differences in family policy between these countries. For example, both provide comparable amounts of universal child benefit;^56^, ^57^ however, in Sweden parents are also offered affordable, subsidised day care^56^ and longer paid parental leave (combined 70 weeks compared with 41 weeks in the UK).^58^ Differences in provision of health care could also contribute to these small remaining differences.^56^, ^57^ However, our results point to mechanisms outside health-care services as being much more important.

The increased child mortality in England relative to Sweden was largely explained by differences in the distribution of birth characteristics between the two countries. Adverse birth characteristics are strongly associated with maternal health. Therefore, the largest reductions in child mortality in England relative to Sweden could be achieved through universal programmes to improve the health of women and reduce health inequalities before and during pregnancy.
