# Supplementary material for: Child mortality in England compared with Sweden: a birth cohort study
Source: Lancet. 2018 May 19;391(10134):2008–18. doi: 10.1016/S0140-6736(18)30670-6 (PMC5958228; doi:10.1016/S0140-6736(18)30670-6)
Supplement: Supplementary appendix [file mmc1.pdf]

THE LANCET

Supplementary appendix

This appendix formed part of the original submission and has been peer reviewed. We post it as supplied by the authors.

Supplement to: Zylbersztejn A, Gilbert R, Hjern A, Wijlaars L, Hardelid P. Child mortality in England compared with Sweden: a birth cohort study. *Lancet* 2018; published online May 3. http://dx.doi.org/10.1016/S0140-6736(18)30670-6.

Appendix A: Deriving and validating the English birth cohort

Developing a nationally representative birth cohort using HES APC data

The English birth cohort was developed by identifying birth admission records in Hospital Episode Statistics Admitted Patient Care data (HES APC, referred to as HES throughout this document). The cohort covered 6,100,404 births in 2003-2012, which accounted for approximately 97% of all singleton live births in England. Birth weight, gestational age and maternal age were missing for 34%, 37% and 37% of records respectively (table 1). Socio-economic status was missing in 58% of records.

For every birth, two records are created in HES – a birth admission record for the baby and a delivery record for the mother.(1) Both records contain additional variables describing the delivery and birth, such as gestational age, birth weight, maternal age, parity and delivery method.(1) Maternal delivery records are often more complete than birth records.(2) Therefore, we enhanced the completeness of risk factors at birth by linking maternal deliveries with baby's birth admissions.(3) As a result, the rate of missing data reduced to 17%, 23%, 3·1%, and 2·5% for birth weight, gestational age, maternal age, and socio-economic status, respectively (table 1).

Table 1 - Percentage of records with missing information about risk factors at birth, before and after linking birth and delivery admissions

Risk factor	HES cohort using information from birth admissions only	HES cohort using information from linked birth and delivery admissions
Birth weight	34%	17%
Gestational age	37%	23%
Maternal age	37%	3·1%
IMD score	58%	2·5%
Sex	0·1%	0·1%

HES=Hospital Episode Statistics, IMD=Index of Multiple Deprivation. IMD score is an indicator of socio-economic status

Validating the birth cohort

To validate the HES birth cohort, we compared child mortality rates based on all births and deaths recorded in HES ("whole cohort"), and based only on births and deaths with complete information on all birth characteristics and socio-economic factors ("complete case cohort"), with national statistics for England and Wales, reported by the Office for National Statistics (ONS).(4,5) Rates in the whole cohort were representative of ONS mortality rates for England and Wales (table 2). However, the rates based on complete case cohort were underestimated compared to the ONS mortality rates, especially in the first week of life.

Table 2 - Comparison of crude mortality rates in birth cohort developed using HES and in England and Wales in 2003-2012

Age at death	HES – whole cohort*	HES - complete case cohort†	England and Wales (ONS)‡
0-1 days	1·59	0·80	1·61
2-6 days	0·59	0·46	0·54
7-27 days	0·67	0·57	0·64
28-364 days	1·34	1·22	1·33
0-364 days	2·60	2·25	2·51

HES=Hospital Episode Statistics, ONS=Office for National Statistics. All data are rates per 1000 singleton live births. *Whole cohort covers all singleton live births in England to resident mothers identified in HES in 2003-2012. †Complete case cohort covers all singleton live births in England to resident mothers identified in HES in 2003-2012 with complete information on birth weight, gestational age, sex, socio economic status (Index of Multiple Deprivation score) and maternal age. ‡Rates for England and Wales were obtained from ONS mortality publications for 2003-2012.(4,5) Mortality rate on days 0-1 in England and Wales was estimated by assuming that approximately ¾ of deaths on days 0-6 occur in the first two days.(6)

Identifying hospitals with high quality of recorded data

Completeness and quality of recorded birth characteristics vary between hospitals, as these variables are collected from local maternity systems in each hospital and are not mandated returns to HES.(1) For example, approximately 20 hospitals have stand-alone maternity systems and do not feed any maternity information into HES.(7,8) To overcome the problem of missing data, some authors recommended using a sub-cohort of hospitals with high completeness of risk factors at birth for the analyses.(9) Therefore, we developed criteria for identifying hospitals with high completeness of gestational age and birth weight, and high quality of linkage with ONS mortality data in an iterative process. The final inclusion criteria for the hospitals were:

- >25% of records with complete birth weight and gestational age per financial year
- >500 births per financial year
- At least 1 infant death per financial year
- At least half of the deaths at age 2 days – 4 years linked to an ONS death record (there are some deaths that are recorded only in HES)
- If there were any deaths at age 28-364 days, at least one had complete information about birth weight and gestational age
- If there were any deaths at age 7-27 days, at least one had complete information about birth weight and gestational age
- If there were any deaths at age 2-6 days, at least one had complete information about birth weight and gestational age

The derived cohort covered 71·0% of all births initially identified in HES. Number of births and deaths excluded at each stage are presented in figure 1.

Figure 1 - Flow chart illustrating steps taken to derive a representative birth cohort based on selected hospitals with “good” quality of recorded data

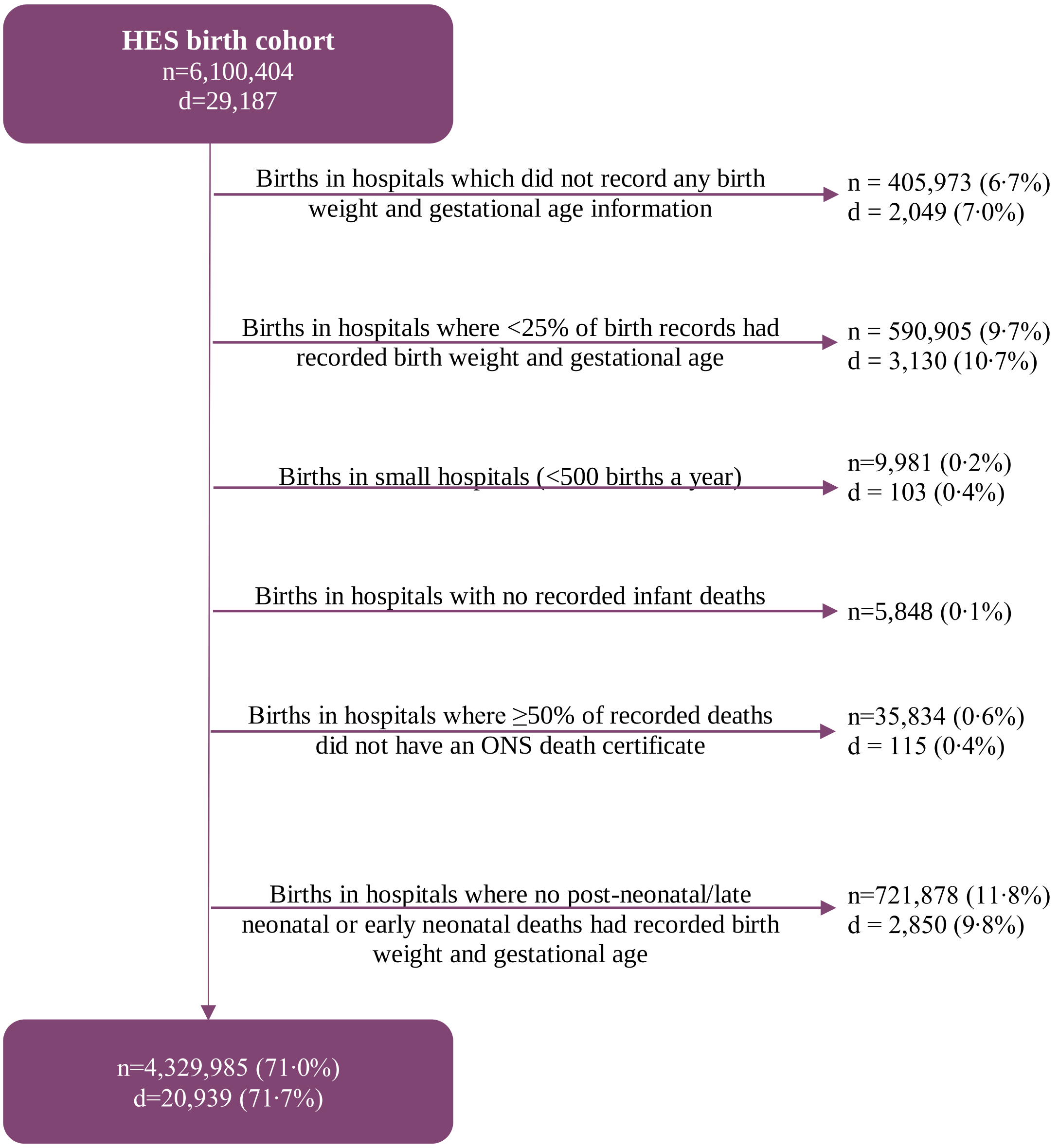

HES=Hospital Episode Statistics, ONS=Office for National Statistics. Numbers of live births (n), deaths (d) excluded at each step are presented. For each exclusion criterion, percentage of all singleton live births and all deaths are shown in brackets.

Validation of the cohort based on births in selected hospitals

To validate the cohort based on births in selected hospitals, we first compared crude mortality rates by age at death in the whole and complete case cohorts, with national statistics for England and Wales published by the ONS.(4,5,10) Rates in the complete case cohort were representative of ONS mortality rates beyond days 0-1 of life (table 3). Therefore, we excluded these early deaths from the analyses. Deaths on 2-6 days, 7-27 days, 28-365 days were slightly underestimated compared to the ONS mortality rates for England and Wales. However, similar underestimation between complete case cohort and the whole population was observed in the Swedish data (table 3).

Table 3 - Comparison of crude infant mortality rates in whole and complete case cohorts of selected hospitals in HES and in England and Wales, and between whole and complete case cohorts in Sweden

Age at death	HES – whole cohort based on selected hospitals	HES – complete case cohort based on selected hospitals	England and Wales (ONS)*	Sweden – whole cohort	Sweden – complete case cohort
0-1 days	1·58	0·83	1·61	0·75	0·67
2-6 days	0·59	0·49	0·54	0·41	0·36
7-27 days	0·70	0·61	0·64	0·44	0·41
28-364 days	1·39	1·28	1·33	0·88	0·85
0-364 days	4·25	3·22	4·12	2·47	2·29

HES=Hospital Episode Statistics, ONS=Office for National Statistics. All data are mortality rates per 1000 live births. *Information for England and Wales was obtained from ONS mortality publications for 2003-2012.(4,5) Mortality rate on days 0-1 in England and Wales was estimated by assuming that approximately ¾ of deaths on days 0-6 occur in the first two days.(6)

We next compared the distribution of birth weight, maternal age and gestational age in the complete case cohort with national statistics for England and Wales published by the ONS.(4,5,10) The distribution of all three risk factors was largely representative of the population of infants in England and Wales apart from births at <24 weeks or weighing <1000g at birth, which were underrepresented (table 4).

Table 4 – Distribution of birth weight, maternal age and gestational age in the complete case cohort of selected hospitals in HES and in England and Wales

	HES - complete case cohort based on selected hospitals	England and Wales (ONS)*
**Birth weight (grams)**		
<1000	0·28%	0·42%
1000-1499	0·47%	0·51%
1500-1999	1·0%	1·0%
2000-2499	3·8%	3·8%
2500-2999	16%	16%
3000-3499	37%	37%
3500-3999	30%	30%
4000+	11%	12%
**Maternal age (years)**		
<20	6·1%	6·3%
20-24	19%	19%
25-29	27%	27%
30-34	28%	29%
35-39	16%	16%
≥40	3·6%	3·6%
**Gestational age (2010-2012) (weeks)**		
< 24	0·0088%	0·085%
24-27	0·23%	0·27%
28-31	0·54%	0·60%
32-36	4·7%	4·7%
37-41	90%	90%
42+	4·5%	4·2%

HES=Hospital Episode Statistics, ONS=Office for National Statistics. All data are % of all singleton live births. *Information for England and Wales was obtained from ONS mortality publications for 2003-2012.(4,5) For gestational age tabulations, we used data from 2010-12, as only for these years the data was consistently coded in the same categories in ONS publications.(10)

Finally, we compared the distribution of risk factors in infants who died and mortality rates by risk factor categories in the HES complete case cohort based on selected hospitals with ONS national statistics for England and Wales. Data from ONS publications was available for birth weight-specific and maternal age-specific mortality at 0-6, 7-27 and 28-364 days in 2003-2012, and for gestation-specific mortality at 0-27 and 28-364 days in 2010-2012. Therefore, to assess the representativeness of the HES complete case cohort based on selected hospitals against ONS national publications we included deaths on days 0-1 of life when calculating rates presented in table 5 (even though mortality on days 0-1 of life was underestimated in the complete case cohort, as shown in table 3).

Birth weight-specific mortality rates were representative for England and Wales at all ages at death, apart from for births weighing <1000g, which were underestimated at 0-6 days and overestimated for deaths beyond first week of life. Similarly, gestation-specific mortality rates were comparable with ONS rates reported for England and Wales for all gestations and ages at death apart from <24 weeks, which were underestimated at 0-27 days and overestimated at 28-364 days. The problem was not present for deaths in infants born at 24-27 weeks' gestation.

In England, birth weight of <500g is most common for births at <24 weeks' gestation and the majority of births at 24-27 weeks' gestation weigh 500-999g at birth (e.g., in 2005, 70% of births at 24-27 weeks' gestation weighed 500-999g, while only 3.3% weighed <500g).(11) Therefore, we assumed that birth weight of 500-999g is equivalent to gestational age of 24-27 weeks. Under this assumption, underreporting of mortality rates for birth weight <1000g was primarily driven by underestimated mortality rates for birth weight <500g (equivalent to gestational age <24 weeks)(11), and mortality rates for a birth weight of 500-999g were representative of rates for England and Wales (similarly to rates for a gestational age of 24-27 weeks).

For maternal age, mortality rates in the complete case cohort based on selected hospitals were underestimated at 0-6 days for all maternal age categories, reflecting underrepresentation of deaths on days 0-1 of life in the complete case cohort compared to England and Wales (table 3). Therefore, we assumed that after excluding deaths on days 0-1 of life, the distribution of maternal age was representative of mothers in England and Wales.

Conclusion

The complete case cohort based on selected hospitals was representative of children in England and Wales after applying additional exclusions. We needed to exclude deaths on days 0-1 as these deaths had high rate of missing data (table 3). Mortality rates for birth weight <1000g and gestational age <24 weeks were highly underestimated for early life mortality and overestimated in the post neonatal period (table 5), while the proportions of births with these characteristics were underestimated (table 4), suggesting that births at borderline viability and deaths around the time of birth were more likely to have missing data. The problem was not present for births at 24-27 weeks, and therefore we assumed that births with birth weight of 500-999g were also representative. Therefore, we excluded births and deaths with gestational age <24 week or birth weight <500g and deaths on days 0-1 of life from the analyses. The remaining cohort was representative of children in England.

Table 5 - Mortality rates by risk factors at birth in the complete case cohort based on selected hospitals and in England and Wales

Risk factor	HES – complete case cohort based on selected hospitals	England and Wales (ONS)*	HES – complete case cohort based on selected hospitals	England and Wales (ONS)*	HES – complete case cohort based on selected hospitals	England and Wales (ONS)*
**Birth weight (g)**	**Early neonatal deaths (0-6 days)**		**Late neonatal deaths (7-27 days)**		**Post neonatal deaths (28-364 days)**	
<1000	150	250	62	48	66	52
1000-1499	30	32	11	12	15	16
1500-1999	12	12	4·0	4·2	8·7	9·1
2000-2499	3·5	3·7	1·8	1·6	4·1	4·1
2500-2999	1·1	1·2	0·62	0·57	1·6	1·6
3000-3499	0·50	0·52	0·28	0·26	0·83	0·81
3500-3999	0·35	0·37	0·16	0·16	0·52	0·51
≥4000	0·44	0·48	0·16	0·17	0·46	0·45
**Maternal age (years)**	**Early neonatal deaths (0-6 days)**		**Late neonatal deaths (7-27 days)**		**Post neonatal deaths (28-364 days)**	
<20	1·7	2·8	0·91	1·0	2·4	2·4
20-24	1·4	2·3	0·70	0·74	1·6	1·7
25-29	1·3	2·1	0·61	0·64	1·2	1·3
30-34	1·2	1·9	0·48	0·51	1·0	1·0
35-39	1·4	2·1	0·57	0·58	1·0	1·1
≥40	1·7	2·7	0·87	0·84	1·5	1·6
**Gestational age (weeks, 2010-2012)**			**Neonatal deaths (0-27 days)**		**Post neonatal deaths (28-364 days)**	
<24			490	860	100	27
24-27			190	180	58	57
28-31			40	38	12	12
32-36			6·3	6·4	3·9	3·7
37-41			0·77	0·74	0·80	0·74
≥42			0·75	0·85	0·56	0·53

HES=Hospital Episode Statistics, ONS=Office for National Statistics. All data are mortality rates per 1000 live births rounded to two significant figures. Mortality rates were calculated based on all infant deaths (i.e. including deaths on days 0-1). *Information for England and Wales was obtained from ONS mortality publications for 2003-2012.(4,5) For gestational age tabulations, we used data from 2010-12 as only for these years the data was consistently coded in the same categories in ONS publications.(10)

Appendix B: Supplementary analyses

Additional results

Table 6 – 10 most commonly recorded diagnoses of congenital anomalies in the English and Swedish birth cohorts

England			Sweden		
**ICD-10 code**	**Description**	**Prevalence**	**ICD-10 code**	**Description**	**Prevalence**
Q21	Congenital malformations of cardiac septa	24·3%	Q21	Congenital malformations of cardiac septa	32·4%
Q25	Congenital malformations of great arteries	22·6%	Q25	Congenital malformations of great arteries	12·9%
Q54	Hypospadias	14·9%	Q54	Hypospadias	11·9%
Q62	Congenital obstructive defects of renal pelvis and congenital malformations of ureter	10·5%	Q62	Congenital obstructive defects of renal pelvis and congenital malformations of ureter	7·0%
Q65	Congenital deformities of hip	7·2%	Q24	Other congenital malformations of heart	6·3%
Q24	Other congenital malformations of heart	5·9%	Q65	Congenital deformities of hip	5·3%
Q31	Congenital malformations of larynx	5·6%	Q90	Down syndrome	5·0%
Q75	Other congenital malformations of skull and face bones	4·7%	Q22	Congenital malformations of pulmonary and tricuspid valves	3·4%
Q04	Congenital hydrocephalus	4·4%	Q35	Cleft palate	3·3%
Q63	Other congenital malformations of kidney	4·1%	Q75	Other congenital malformations of skull and face bones	3·2%

ICD-10= International Statistical Classification of Diseases and Related Health Problems 10th Revision. Children could have more than one ICD-10 code recorded. Prevalence was calculated as % of all children with at least one congenital anomaly.

Table 7 – Unadjusted and adjusted Cox proportional hazards models for all-cause mortality at 2-27 days in England relative to Sweden

	Model 1	Model 2	Model 3	Model 4
**Country**				
England	1·66 (1·53, 1·81)	1·37 (1·26, 1·48)	1·15 (1·06, 1·25)	1·13 (1·04, 1·23)
Sweden (baseline)	1	1	1	1
**Birth weight (grams)**				
500-999		31·3 (24·8, 39·5)	16·4 (13·0, 20·6)	15·5 (12·3, 19·6)
1000-1499		11·9 (9·6, 14·7)	7·7 (6·2, 9·5)	7·3 (5·9, 9·1)
1500-2499		6·0 (5·3, 6·9)	5·2 (4·6, 6·0)	5·0 (4·4, 5·7)
2500-3499		1·82 (1·66, 2·00)	1·81 (1·65, 1·98)	1·77 (1·61, 1·94)
≥3500 (baseline)		1	1	1
**Gestational age (weeks)**				
24-27		15·4 (12·3, 19·2)	7·6 (6·1, 9·5)	7·8 (6·2, 9·7)
28-31		5·5 (4·5, 6·7)	3·89 (3·21, 4·72)	3·95 (3·26, 4·80)
32-34		3·39 (2·91, 3·94)	2·91 (2·50, 3·39)	2·95 (2·54, 3·43)
35-36		2·70 (2·38, 3·07)	2·45 (2·16, 2·78)	2·46 (2·17, 2·80)
37-38		1·62 (1·48, 1·76)	1·53 (1·40, 1·67)	1·53 (1·40, 1·68)
≥39 (baseline)		1	1	1
**Sex**				
Boy		1·27 (1·20, 1·35)	1·19 (1·13, 1·26)	1·19 (1·13, 1·26)
Girl (baseline)		1	1	1
**Congenital anomaly**				
Yes			7·2 (6·7, 7·7)	7·1 (6·6, 7·7)
No (baseline)			1	1
**Maternal age (years)**				
<20				1·23 (1·09, 1·38)
20-24				1·14 (1·04, 1·24)
25-29				1·10 (1·02, 1·19)
30-34 (baseline)				1
35-39				1·06 (0·97, 1·16)
≥40				1·32 (1·15, 1·52)
**Quintile of socio-economic status**				
Q1: most deprived				1·24 (1·13, 1·36)
Q2				1·11 (1·01, 1·22)
Q3				1·00 (0·91, 1·11)
Q4				0·94 (0·85, 1·04)
Q5: least deprived (baseline)				1

Data are adjusted hazard ratios (95% confidence intervals). Each column represents a separate Cox proportional hazards model. Data were adjusted for country (model 1), further adjusted for birth weight, gestational age and sex (model 2), for congenital anomalies (model 3) and for maternal age and quintile of socio-economic status (model 4).

Table 8 – Unadjusted and adjusted Cox proportional hazards models for all-cause mortality at 28-364 days in England relative to Sweden

	Model 1	Model 2	Model 3	Model 4
**Country**				
England	1·59 (1·47, 1·71)	1·32 (1·22, 1·42)	1·19 (1·10, 1·28)	1·12 (1·04, 1·21)
Sweden (baseline)	1	1	1	1
**Birth weight (grams)**				
500-1500		27·3 (22·8, 32·8)	11·7 (9·7, 14·0)	10·4 (8·7, 12·6)
1500-2499		7·0 (6·2, 7·8)	5·3 (4·7, 5·9)	4·7 (4·3, 5·3)
2500-3499		2·00 (1·85, 2·15)	1·94 (1·80, 2·09)	1·84 (1·71, 1·98)
≥3500 (baseline)		1	1	1
**Gestational age (weeks)**				
24-32		3·09 (2·60, 3·67)	1·58 (1·33, 1·89)	1·65 (1·38, 1·97)
32-34		1·63 (1·42, 1·88)	1·33 (1·15, 1·52)	1·38 (1·20, 1·59)
35-36		1·92 (1·72, 2·14)	1·63 (1·46, 1·82)	1·66 (1·49, 1·85)
37-38		1·53 (1·43, 1·64)	1·39 (1·30, 1·50)	1·41 (1·32, 1·52)
≥39 (baseline)		1	1	1
**Sex**				
Boy		1·33 (1·26, 1·40)	1·18 (1·12, 1·24)	1·17 (1·11, 1·24)
Girl (baseline)		1	1	1
**Congenital anomaly**				
Yes			15·4 (14·5, 16·3)	15·2 (14·4, 16·2)
No (baseline)			1	1
**Maternal age (years)**				
<20				1·72 (1·56, 1·90)
20-24				1·32 (1·22, 1·42)
25-29				1·10 (1·02, 1·19)
30-34 (baseline)				1
35-39				0·99 (0·90, 1·08)
≥40				1·20 (1·05, 1·38)
**Quintile of socio-economic status**				
Q1: most deprived				1·66 (1·52, 1·81)
Q2				1·49 (1·36, 1·64)
Q3				1·14 (1·04, 1·26)
Q4				1·12 (1·01, 1·24)
Q5: least deprived (baseline)				1

Data are adjusted hazard ratios (95% confidence intervals). Each column represents a separate Cox proportional hazards model. Data were adjusted for country (model 1), further adjusted for birth weight, gestational age and sex (model 2), for congenital anomalies (model 3) and for maternal age and quintile of socio-economic status (model 4).

Table 9 - Unadjusted and adjusted Cox proportional hazards models for all-cause mortality at 1-4 years in England relative to Sweden

	Model 1	Model 2	Model 3	Model 4
**Country**				
England	1·27 (1·15, 1·40)	1·14 (1·03, 1·26)	1·10 (1·00, 1·22)	1·06 (0·96, 1·18)
Sweden (baseline)	1	1	1	1
**Birth weight (grams)**				
500-1500		10·7 (8·3, 13·7)	3·04 (2·36, 3·93)	2·86 (2·22, 3·69)
1500-2499		4·3 (3·63, 5·0)	3·12 (2·67, 3·66)	2·92 (2·49, 3·43)
2500-3499		1·53 (1·40, 1·67)	1·48 (1·36, 1·62)	1·43 (1·31, 1·57)
≥3500 (baseline)		1	1	1
**Gestational age (weeks)**				
24-37		1·05 (0·89, 1·23)	0·84 (0·71, 0·99)	0·86 (0·73, 1·02)
37-38		1·18 (1·07, 1·29)	1·07 (0·97, 1·18)	1·08 (0·98, 1·20)
≥39 (baseline)		1	1	1
**Sex**				
Boy		1·18 (1·10, 1·28)	1·05 (0·97, 1·13)	1·04 (0·97, 1·13)
Girl (baseline)		1	1	1
**Congenital anomaly**				
Yes			17·1 (15·8, 18·6)	17·1 (15·7, 18·6)
No (baseline)			1	1
**Maternal age (years)**				
<20				1·26 (1·07, 1·47)
20-24				1·25 (1·12, 1·40)
25-29				1·01 (0·91, 1·12)
30-34 (baseline)				1
35-39				0·92 (0·81, 1·05)
≥40				0·92 (0·74, 1·15)
**Quintile of socio-economic status**				
Q1: most deprived				1·39 (1·23, 1·58)
Q2				1·29 (1·13, 1·47)
Q3				1·20 (1·05, 1·37)
Q4				1·10 (0·96, 1·26)
Q5: least deprived (baseline)				1

Data are adjusted hazard ratios (95% confidence intervals). Each column represents a separate Cox proportional hazards model. Data were adjusted for country (model 1), further adjusted for birth weight, gestational age and sex (model 2), for congenital anomalies (model 3) and for maternal age and quintile of socio-economic status (model 4).

Sensitivity analyses

Table 10 – Unadjusted and adjusted Cox proportional hazards models for all-cause mortality at 2-27 days in England relative to Sweden using an indicator of severe congenital anomalies

	Model 1	Model 2	Model 3	Model 4
**Country**				
England	1·66 (1·53, 1·81)	1·37 (1·26, 1·48)	1·17 (1·07, 1·27)	1·14 (1·05, 1·24)
Sweden (baseline)	1	1	1	1
**Birth weight (grams)**				
500-999		31·3 (24·8, 39·5)	18·4 (14·6, 23·2)	17·5 (13·9, 22·1)
1000-1499		11·9 (9·6, 14·7)	8·5 (6·9, 10·4)	8·1 (6·6, 10·0)
1500-2499		6·0 (5·3, 6·9)	5·2 (4·5, 5·9)	5·0 (4·4, 5·7)
2500-3499		1·82 (1·66, 2·00)	1·80 (1·64, 1·97)	1·76 (1·61, 1·93)
≥3500 (baseline)		1	1	1
**Gestational age (weeks)**				
24-27		15·4 (12·3, 19·2)	10·8 (8·7, 13·4)	11·0 (8·8, 13·6)
28-31		5·5 (4·5, 6·7)	4·6 (3·80, 5·6)	4·7 (3·86, 5·7)
32-34		15·4 (12·3, 19·2)	10·8 (8·7, 13·4)	11·0 (8·8, 13·6)
35-36		2·70 (2·38, 3·07)	2·45 (2·15, 2·78)	2·46 (2·17, 2·79)
37-38		1·62 (1·48, 1·76)	1·53 (1·40, 1·67)	1·53 (1·40, 1·67)
≥39 (baseline)		1	1	1
**Sex**				
Boy		1·27 (1·20, 1·35)	1·19 (1·12, 1·26)	1·18 (1·12, 1·25)
Girl (baseline)		1	1	1
**Severe congenital anomaly**				
Yes			9·4 (8·8, 10·1)	9·4 (8·7, 10·0)
No (baseline)			1	1
**Maternal age (years)**				
<20				1·22 (1·08, 1·37)
20-24				1·15 (1·05, 1·25)
25-29				1·11 (1·02, 1·20)
30-34 (baseline)				1
35-39				1·04 (0·95, 1·14)
≥40				1·25 (1·09, 1·43)
**Quintile of socio-economic status**				
Q1: most deprived				1·20 (1·09, 1·31)
Q2				1·09 (0·99, 1·19)
Q3				0·98 (0·89, 1·09)
Q4				0·95 (0·86, 1·05)
Q5: least deprived (baseline)				1

Data are adjusted hazard ratios (95% confidence intervals). Each column represents a separate Cox proportional hazards model. Data were adjusted for country (model 1), further adjusted for birth weight, gestational age and sex (model 2), for severe congenital anomalies (model 3) and for maternal age and quintile of socio-economic status (model 4).

Table 11 - Unadjusted and adjusted Cox proportional hazards models for all-cause mortality at 28-364 days in England relative to Sweden using an indicator of severe congenital anomalies

	Model 1	Model 2	Model 3	Model 4
**Country**				
England	1·59 (1·47, 1·71)	1·32 (1·22, 1·42)	1·17 (1·09, 1·26)	1·10 (1·02, 1·19)
Sweden (baseline)	1	1	1	1
**Birth weight (grams)**				
500-1500		27·3 (22·8, 32·8)	13·8 (11·5, 16·6)	12·4 (10·3, 14·9)
1500-2499		7·0 (6·2, 7·8)	5·3 (4·7, 5·9)	4·8 (4·3, 5·3)
2500-3499		2·00 (1·85, 2·15)	1·94 (1·80, 2·09)	1·84 (1·71, 1·98)
≥3500 (baseline)		1	1	1
**Gestational age (weeks)**				
24-32		3·09 (2·60, 3·67)	2·10 (1·77, 2·50)	2·19 (1·84, 2·60)
32-34		1·63 (1·42, 1·88)	1·37 (1·19, 1·58)	1·43 (1·24, 1·65)
35-36		1·92 (1·72, 2·14)	1·61 (1·45, 1·80)	1·65 (1·48, 1·84)
37-38		1·53 (1·43, 1·64)	1·38 (1·29, 1·48)	1·40 (1·31, 1·50)
≥39 (baseline)		1	1	1
**Sex**				
Boy		1·33 (1·26, 1·40)	1·20 (1·14, 1·27)	1·20 (1·14, 1·26)
Girl (baseline)		1	1	1
**Severe congenital anomaly**				
Yes			19·6 (18·4, 20·8)	19·3 (18·2, 20·5)
No (baseline)			1	1
**Maternal age (years)**				
<20				1·70 (1·54, 1·87)
20-24				1·33 (1·23, 1·43)
25-29				1·11 (1·03, 1·20)
30-34 (baseline)				1
35-39				0·97 (0·89, 1·07)
≥40				1·15 (1·00, 1·32)
**Quintile of socio-economic status**				
Q1: most deprived				1·62 (1·48, 1·77)
Q2				1·47 (1·34, 1·61)
Q3				1·13 (1·02, 1·24)
Q4				1·12 (1·02, 1·24)
Q5: least deprived (baseline)				1

Data are adjusted hazard ratios (95% confidence intervals). Each column represents a separate Cox proportional hazards model. Data were adjusted for country (model 1), further adjusted for birth weight, gestational age and sex (model 2), for severe congenital anomalies (model 3) and for maternal age and quintile of socio-economic status (model 4).

Table 12 Unadjusted and adjusted Cox proportional hazards models for all-cause mortality at 1-4 years in England relative to Sweden using an indicator of severe congenital anomalies

	Model 1	Model 2	Model 3	Model 4
**Country**				
England	1·27 (1·15, 1·40)	1·14 (1·03, 1·26)	1·06 (0·96, 1·17)	1·03 (0·93, 1·13)
Sweden (baseline)	1	1	1	1
**Birth weight (grams)**				
500-1500		10·7 (8·3, 13·7)	4·2 (3·27, 5·4)	3·96 (3·08, 5·1)
1500-2499		4·3 (3·63, 5·0)	3·05 (2·61, 3·57)	2·85 (2·43, 3·34)
2500-3499		1·53 (1·40, 1·67)	1·47 (1·35, 1·61)	1·42 (1·30, 1·56)
≥3500 (baseline)		1	1	1
**Gestational age (weeks)**				
24-37		1·05 (0·89, 1·23)	0·83 (0·71, 0·98)	0·85 (0·72, 1·01)
37-38		1·18 (1·07, 1·29)	1·04 (0·94, 1·15)	1·05 (0·96, 1·16)
≥39 (baseline)		1	1	1
**Sex**				
Boy		1·18 (1·10, 1·28)	1·06 (0·98, 1·15)	1·06 (0·98, 1·14)
Girl (baseline)		1	1	1
**Severe congenital anomaly**				
Yes			27·0 (24·8, 29·4)	26·9 (24·7, 29·3)
No (baseline)			1	1
**Maternal age (years)**				
<20				1·24 (1·06, 1·45)
20-24				1·25 (1·12, 1·40)
25-29				1·01 (0·91, 1·13)
30-34 (baseline)				1
35-39				0·92 (0·81, 1·04)
≥40				0·88 (0·70, 1·10)
**Quintile of socio-economic status**				
Q1: most deprived				1·37 (1·21, 1·56)
Q2				1·28 (1·12, 1·45)
Q3				1·19 (1·05, 1·36)
Q4				1·10 (0·96, 1·26)
Q5: least deprived (baseline)				1

Data are adjusted hazard ratios (95% confidence intervals). Each column represents a separate Cox proportional hazards model. Data were adjusted for country (model 1), further adjusted for birth weight, gestational age and sex (model 2), for severe congenital anomalies (model 3) and for maternal age and quintile of socio-economic status (model 4).

Table 13 – Unadjusted and adjusted Cox proportional hazards models for all-cause mortality at 2-27 days in England relative to Sweden including an effect modification term with time for congenital anomalies indictor

	Model 1	Model 2	Model 3	Model 4
**Country**				
England	1·66 (1·53, 1·81)	1·37 (1·26, 1·48)	1·15 (1·06, 1·25)	1·13 (1·04, 1·23)
Sweden (baseline)	1	1	1	1
**Birth weight (grams)**				
500-999		31·3 (24·8, 39·5)	16·4 (13·0, 20·7)	15·6 (12·3, 19·6)
1000-1499		11·9 (9·6, 14·7)	7·7 (6·2, 9·5)	7·3 (5·9, 9·1)
1500-2499		6·0 (5·3, 6·9)	5·2 (4·6, 6·0)	5·0 (4·4, 5·7)
2500-3499		1·82 (1·66, 2·00)	1·81 (1·65, 1·98)	1·77 (1·61, 1·94)
≥3500 (baseline)		1	1	1
**Gestational age (weeks)**				
24-27		15·4 (12·3, 19·2)	7·6 (6·1, 9·5)	7·8 (6·2, 9·7)
28-31		5·5 (4·5, 6·7)	3·89 (3·20, 4·7)	3·95 (3·25, 4·8)
32-34		3·39 (2·91, 3·94)	2·91 (2·50, 3·39)	2·95 (2·54, 3·43)
35-36		2·70 (2·38, 3·07)	2·45 (2·16, 2·78)	2·46 (2·17, 2·80)
37-38		1·62 (1·48, 1·76)	1·53 (1·40, 1·67)	1·53 (1·40, 1·68)
≥39 (baseline)		1	1	1
**Sex**				
Boy		1·27 (1·20, 1·35)	1·19 (1·13, 1·26)	1·19 (1·13, 1·26)
Girl (baseline)		1	1	1
**Congenital anomaly**				
Yes			5·2 (4·7, 5·8)	5·2 (4·7, 5·8)
effect with time (days)			1·03 (1·02, 1·04)	1·03 (1·02, 1·04)
No (baseline)			1	1
**Maternal age (years)**				
<20				1·23 (1·09, 1·38)
20-24				1·14 (1·04, 1·24)
25-29				1·10 (1·02, 1·19)
30-34 (baseline)				1
35-39				1·06 (0·97, 1·16)
≥40				1·32 (1·15, 1·52)
**Quintile of socio-economic status**				
Q1: most deprived				1·24 (1·13, 1·36)
Q2				1·11 (1·01, 1·22)
Q3				1·00 (0·91, 1·11)
Q4				0·94 (0·85, 1·04)
Q5: least deprived (baseline)				1

Data are adjusted hazard ratios (95% confidence intervals). Each column represents a separate Cox proportional hazards model. Data were adjusted for country (model 1), further adjusted for birth weight, gestational age and sex (model 2), for congenital anomalies including an effect modification term with age (model 3) and for maternal age and quintile of socio-economic status (model 4).

Table 14 - Unadjusted and adjusted Cox proportional hazards models for all-cause mortality at 28-364 days in England relative to Sweden including an effect modification term with time for congenital anomalies indicator

	Model 1	Model 2	Model 3	Model 4
**Country**				
England	1·59 (1·47, 1·71)	1·32 (1·22, 1·42)	1·19 (1·10, 1·28)	1·12 (1·04, 1·21)
Sweden (baseline)	1	1	1	1
**Birth weight (grams)**				
500-1500		27·3 (22·8, 32·8)	11·7 (9·7, 14·1)	10·4 (8·7, 12·6)
1500-2499		7·0 (6·2, 7·8)	5·3 (4·7, 5·9)	4·7 (4·3, 5·3)
2500-3499		2·00 (1·85, 2·15)	1·94 (1·80, 2·09)	1·84 (1·71, 1·98)
≥3500 (baseline)		1	1	1
**Gestational age (weeks)**				
24-32		3·09 (2·60, 3·67)	1·58 (1·33, 1·89)	1·65 (1·38, 1·97)
32-34		1·63 (1·42, 1·88)	1·33 (1·15, 1·52)	1·38 (1·20, 1·59)
35-36		1·92 (1·72, 2·14)	1·63 (1·46, 1·82)	1·66 (1·49, 1·85)
37-38		1·53 (1·43, 1·64)	1·39 (1·30, 1·50)	1·41 (1·32, 1·52)
≥39 (baseline)		1	1	1
**Sex**				
Boy		1·33 (1·26, 1·40)	1·18 (1·12, 1·24)	1·17 (1·11, 1·24)
Girl (baseline)		1	1	1
**Congenital anomaly**				
Yes			14·6 (13·3, 16·1)	14·4 (13·0, 15·9)
effect with time (months)			1·01 (0·99, 1·03)	1·05 (0·98, 1·13)
No (baseline)			1	1
**Maternal age (years)**				
<20				1·72 (1·56, 1·90)
20-24				1·32 (1·22, 1·42)
25-29				1·10 (1·02, 1·19)
30-34 (baseline)				1
35-39				0·99 (0·90, 1·08)
≥40				1·20 (1·05, 1·38)
**Quintile of socio-economic status**				
Q1: most deprived				1·66 (1·52, 1·81)
Q2				1·49 (1·36, 1·64)
Q3				1·14 (1·04, 1·26)
Q4				1·12 (1·01, 1·24)
Q5: least deprived (baseline)				1

Data are adjusted hazard ratios (95% confidence intervals). Each column represents a separate Cox proportional hazards model. Data were adjusted for country (model 1), further adjusted for birth weight, gestational age and sex (model 2), for congenital anomalies including an effect modification term with age (model 3) and for maternal age and quintile of socio-economic status (model 4).

Table 15 – Unadjusted and adjusted Cox proportional hazards models for all-cause mortality at 1-4 years in England relative to Sweden including an effect modification term with time for congenital anomalies indicator

	Model 1	Model 2	Model 3	Model 4
**Country**				
England	1·27 (1·15, 1·40)	1·14 (1·03, 1·26)	1·10 (1·00, 1·22)	1·06 (0·96, 1·18)
Sweden (baseline)	1	1	1	1
**Birth weight (grams)**				
500-1500		10·7 (8·3, 13·7)	3·04 (2·36, 3·92)	2·86 (2·22, 3·68)
1500-2499		4·3 (3·63, 5·0)	3·12 (2·67, 3·66)	2·92 (2·49, 3·42)
2500-3499		1·53 (1·40, 1·67)	1·48 (1·36, 1·62)	1·43 (1·31, 1·57)
≥3500 (baseline)		1	1	1
**Gestational age (weeks)**				
24-37		1·05 (0·89, 1·23)	0·84 (0·71, 0·99)	0·86 (0·73, 1·02)
37-38		1·18 (1·07, 1·29)	1·07 (0·97, 1·18)	1·08 (0·98, 1·20)
≥39 (baseline)		1	1	1
**Sex**				
Boy		1·18 (1·10, 1·28)	1·05 (0·97, 1·13)	1·04 (0·97, 1·13)
Girl (baseline)		1	1	1
**Congenital anomaly**				
Yes			26·1 (21·6, 31·5)	26·0 (21·5, 31·4)
effect with time (years)			0·83 (0·77, 0·90)	0·83 (0·77, 0·90)
No (baseline)			1	1
**Maternal age (years)**				
<20				1·26 (1·07, 1·47)
20-24				1·25 (1·12, 1·40)
25-29				1·01 (0·91, 1·12)
30-34 (baseline)				1
35-39				0·92 (0·81, 1·05)
≥40				0·92 (0·74, 1·15)
**Quintile of socio-economic status**				
Q1: most deprived				1·39 (1·22, 1·58)
Q2				1·29 (1·13, 1·47)
Q3				1·20 (1·05, 1·37)
Q4				1·10 (0·96, 1·26)
Q5: least deprived (baseline)				1

Data are adjusted hazard ratios (95% confidence intervals). Each column represents a separate Cox proportional hazards model. Data were adjusted for country (model 1), further adjusted for birth weight, gestational age and sex (model 2), for congenital anomalies including an effect modification term with age (model 3) and for maternal age and quintile of socio-economic status (model 4).

References

(1) Herbert A, Wijlaars L, Zylbersztejn A, Cromwell D, Hardelid P. Data Resource Profile: Hospital Episode Statistics Admitted Patient Care (HES APC). Int J Epidemiol 2017 Mar 15.

(2) Ghosh RE, Ashworth DC, Hansell AL, Garwood K, Elliott P, Toledano MB. Routinely collected English birth data sets: comparisons and recommendations for reproductive epidemiology. Arch Dis Child Fetal Neonatal Ed 2016 Feb 2.

(3) Harron K, Gilbert R, Cromwell D, van der Meulen J. Linking Data for Mothers and Babies in De-Identified Electronic Health Data. PLoS One 2016 Oct 20;11(10):e0164667.

(4) Office for National Statistics. Birth cohort tables for infant deaths, 2007-2012. 2007-2012.

(5) Office for National Statistics. Mortality Statistics: Childhood, infant and perinatal, England and Wales (Series DH3). 2003-2007.

(6) Cortina Borja M. email communication. 2017.

(7) Dattani N, Datta-Nemdharry P, Macfarlane A. Linking maternity data for England, 2005-06: methods and data quality. Health Stat Q 2011 Spring;(49):53-79. doi(49):53-79.

(8) Dattani N, Datta-Nemdharry P, Macfarlane A. Linking maternity data for England 2007: methods and data quality. Health Stat Q 2012 Spring;(53)(53):4-21.

(9) Murray J, Saxena S, Modi N, Majeed A, Aylin P, Bottle A, et al. Quality of routine hospital birth records and the feasibility of their use for creating birth cohorts. J Public Health (Oxf) 2013 Jun;35(2):298-307.

(10) Office for National Statistics. Gestation-specific Infant Mortality in England and Wales. 2007-2012.

(11) Moser K, Hilder L. Assessing quality of NHS Numbers for Babies data and providing gestational age statistics. Health Stat Q 2008 Spring;(37)(37):15-23.
